# Recent developments in gene therapy for Parkinson’s disease

**DOI:** 10.1016/j.ymthe.2025.03.030

**Published:** 2025-03-22

**Authors:** Sandor Szunyogh, Emily Carroll, Richard Wade-Martins

**Affiliations:** 1 Oxford Parkinson’s Disease Centre, Department of Physiology, Anatomy and Genetics, University of Oxford, Oxford OX1 3QX, UK; 2 Kavli Institute for Nanoscience Discovery, University of Oxford, Dorothy Crowfoot Hodgkin Building, South Parks Road, Oxford OX1 3QU, UK

**Keywords:** Parkinson’s disease, gene therapy, AAV, HSV-1, lentivirus, dopamine neurons, growth factor delivery, neuromodulation

## Abstract

Parkinson’s disease (PD) is a progressive, neurodegenerative disorder for which there is currently no cure. Gene therapy has emerged as a novel approach offering renewed hope for the development of treatments that meaningfully alter the course of the disease. In this review, we explore various gene therapy strategies currently being developed targeting key aspects of PD pathogenesis: the restoration of the dopamine system by delivering genes involved in dopamine biosynthesis, reinforcing the inhibitory signaling pathways through glutamic acid decarboxylase (*GAD*) delivery to increase GABA production, enhancing neuronal survival and development by introducing various neurotrophic factors, delivery of genes to complement recessive familial PD mutations to correct mitochondrial dysfunction, restoring lysosomal function through delivery of *GBA1* to increase glucocerebrosidase (GCase) activity, and reducing α-synuclein levels by reducing or silencing *SNCA* expression. Despite promising early work, challenges remain in developing safe, effective, and long-lasting gene therapies. Key considerations include optimizing viral vectors for targeted delivery, achieving controlled and sustained gene expression using different promoters, minimizing immune responses, and increasing transgene delivery capacity. Future prospects may involve combinatory strategies targeting multiple pathways, such as multi-gene constructs delivered via high-capacity viral systems.

## Graphical abstract

**Figure:**
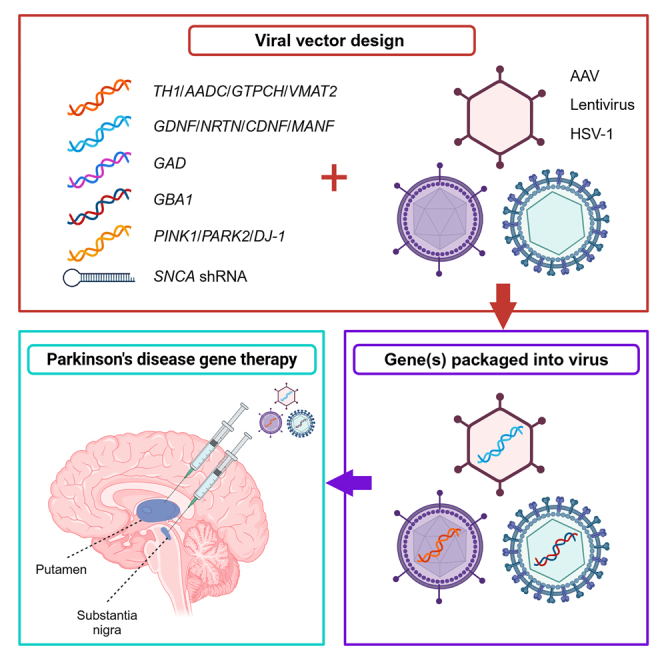

## Teaser

Wade-Martins and colleagues outline current gene therapy approaches under development for the treatment of Parkinson’s disease (PD). The authors discuss the progress of several strategies targeting different aspects of PD pathophysiology and highlight remaining challenges in vector design and delivery.

## Introduction

Parkinson’s disease (PD) is a progressive, neurodegenerative disorder which affects approximately 8.5 million people globally,^1^ and is estimated to rise to 10 million by 2025, far exceeding earlier estimations.^2^ Clinically, the disorder is diagnosed primarily by the presence of motor symptoms including bradykinesia, postural instability, resting tremor, and rigidity; however, many patients experience additional non-motor symptoms including impaired cognition, autonomic dysfunction, and sleep disturbances.^3^^,^^4^ At the cellular level, PD is characterized predominantly by the loss of dopaminergic neurons in the substantia nigra. The pathological hallmark of the disease is the presence of Lewy bodies composed of aggregated proteins including α-synuclein, yet how these aggregates contribute to neurodegeneration is still unclear.^4^

There is currently no cure for PD; current treatments aim to manage symptoms and improve quality of life. Since its development in the 1960s, the dopamine precursor, levodopa, remains the most effective treatment for the management of motor symptoms by increasing dopamine production in the brain.^5^ However, long-term treatment with levodopa will eventually result in adverse effects including dyskinesia, motor fluctuations, and hallucinations.^6^^,^^7^ Additionally, treatment options that target the non-motor symptoms of PD, which significantly impact quality of life, remain limited.^8^ There is increasing demand for interventions that not only provide symptomatic relief but directly target the underlying pathophysiology to slow or halt disease progression.

Gene therapy offers renewed hope for the development of new treatments that significantly alter the course of the disease. The term “gene therapy” may be broadly defined as an intervention that alters the expression of a gene (or genes) to treat, prevent, or cure a disease. This umbrella term encompasses several different approaches including: (1) gene complementation, in which a dysfunctional gene is complemented with a functional copy, (2) gene silencing, whereby expression of a dysfunctional gene resulting in toxicity is reduced or silenced, (3) gene supplementation, the introduction of a new gene into a cell in order to support cellular function, and (4) gene editing, which refers to the permanent modification of an existing gene to correct a genetic mutation leading to disease.^9^

The recent success of gene therapy approaches designed for the treatment of spinal muscular atrophy has fueled interest in exploring its potential for other neurodegenerative diseases.^10^ While only a small percentage of PD cases are directly attributed to a specific genetic mutation,^11^ gene therapy that targets underlying disease pathways offer the potential for more widely applicable treatments. Gene therapies currently under development for PD aim to increase dopamine production, restore imbalances in network excitability, survival of dopamine neurons, and counteract genetic mutations that directly contribute to PD risk or decrease α-synuclein levels.

In this review, we aim to summarize the current gene therapy landscape for PD. We provide an overview of viral vectors used for gene delivery and discuss the rationale and progress of gene therapy targeting different aspects of PD pathogenesis. Furthermore, we discuss the challenges facing gene therapy development for PD and identify strategies to improve the success of these approaches in the clinic.

## Viral vectors for gene delivery

The effective delivery of genetic material to the target cell requires a gene delivery vehicle, termed a vector, which can be either viral or non-viral in origin. Viruses provide an attractive vector system due to their capability to carry transgenes, protecting them from biological degradation, and delivering them efficiently to cells. More recently, non-viral vectors, including cationic lipids and polymers, have been developed to overcome issues with inflammatory and immune reactions triggered by viral vectors. However, the development of these non-viral vectors is still in its relative infancy; therefore we focus on discussing viral vectors, which currently remain the most frequently used vector system for gene delivery.^12^

Several different viral vectors have been used in different model systems including adenovirus, lentivirus, γ-retrovirus, adeno-associated virus (AAV), herpes simplex virus (HSV), vaccinia virus, measles virus, vesicular stomatitis virus, polio virus, and reovirus.^13^ The safety and efficiency of adenovirus, lentivirus, AAV and HSV in humans have been shown extensively and these four viral vectors and their variants dominate the clinical landscape.^14^ Each viral delivery system has unique properties, which can be advantageous depending on the clinical application.^14^ A key difference is the maximum size of the transgene cassette, which ranges from ∼5 kb (AAV) to ∼150 kb (HSV amplicon), which impacts the complexity of the transgene cargo ranging from a single cDNA transcript variant with a heterologous promoter, to a whole genomic region including introns and exons utilizing alternative splicing and regulated by the endogenous promoter usage.^15^ Lentiviral vectors are most commonly used for *ex vivo* gene and cell therapies for editing in hematopoietic stem/progenitor cells,^16^ while different AAV serotypes show selective tropism for specific tissues/cell types *in vivo.*^17^ The integration of the transgene is also an important aspect: retroviruses (including lentivirus) provide long-term expression by stable integration into the host cell’s genome, whereas adenovirus, AAV, and HSV persist by forming episomes in the cell, often resulting in transient expression.

While each viral delivery system comes with its own challenges to consider, including the size of the transgene cassette, the severity of immune response, and the potential for insertional mutagenesis, efforts to enhance the efficacy and safety of these systems have been made and are still ongoing.^16^^,^^18^^,^^19^

## Gene therapy approaches for PD

### Restoring the dopaminergic system

In PD, the selective loss of dopaminergic neurons in the substantia nigra leads to dopamine depletion in the striatum and motor impairment. Dopamine replacement therapy is a treatment aimed at increasing dopamine levels in the brain by supplementation with the dopamine precursor levodopa, which can cross the blood-brain barrier (BBB) and is converted to dopamine by the enzyme dopa decarboxylase. To prevent the conversion of levodopa to dopamine in the periphery, levodopa is administered together with a dopa decarboxylase inhibitor (carbidopa or benserazide). These inhibitors do not cross the BBB, allowing levodopa to be broken down to dopamine in the brain and exert its therapeutic effects.^20^

**Figure 1:**
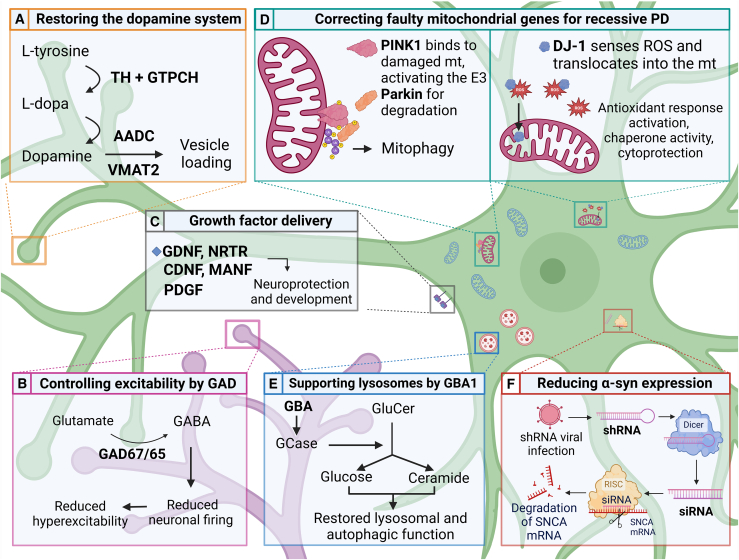
Summary of gene therapy approaches targeting PD pathophysiology (A) Restoring the dopamine system through delivery of genes involved in dopamine biosynthesis and dopamine release, including tyrosine hydroxylase (*TH*), GTP cyclohydrolase I (*GTPCH*), aromatic amino acid decarboxylase (*AADC*), and vesicular monoamine transporter 2 (*VMAT2*). (B) Reinforcing inhibitory pathways in the basal ganglia through delivery of genes encoding glutamic acid decarboxylase isoforms (*GAD65/67*), which converts glutamate to GABA to reduce neuronal firing. (C) Promoting survival and development of dopaminergic neurons through the delivery of genes encoding growth factors including glial cell line-derived neurotrophic factor (*GDNF*), neurturin (*NRTN*), cerebral dopaminergic neurotrophic factor (*CDNF*), mesencephalic astrocyte-derived neurotrophic factor (*MANF*), and platelet-derived growth factor (*PDGF*). (D) Delivery of genes encoding *PINK1*, *PRKN*, and *DJ-1* to address mitochondrial dysfunction resulting from recessive familial PD mutations. (E) Supporting lysosomal function through the delivery of *GBA1* to increase glucocerebrosidase activity (GCase) and promote the breakdown of glucosylceramide (GluCer) to glucose and ceramide in the lysosome. (F) Reducing α-synuclein expression through delivery of short hairpin RNA (shRNA). shRNA is processed by dicer to form short interfering RNA (siRNA), which targets the *SNCA* gene and promotes mRNA degradation.

Gene therapy approaches are being explored for idiopathic PD, aimed at replacing dopamine by introducing genes required for dopamine synthesis at a specific site rather than a systemic treatment (Figure 1A). These genes are tyrosine hydroxylase (*TH*), converting tyrosine to l-3,4-dihydroxyphenylalanine (l-DOPA), aromatic amino acid decarboxylase (*AADC*), converting l-DOPA to dopamine, and GTP cyclohydrolase I (*GTPCH*), which is the rate-limiting enzyme in the synthesis of tetrahydrobiopterin, a cofactor of TH. It has been shown that these three catecholaminergic synthetic enzymes on a single lentiviral vector can achieve functional improvement in the 6-hydroxydopamine (6-OHDA) animal model of PD.^21^ In another study, the vesicular monoamine transporter (*VMAT2*) gene was added to form a four-gene vector in a helper virus-free HSV type 1 vector to control not only dopamine production, but also dopamine release.^22^ VMAT2 transports dopamine into synaptic vesicles supporting regulated vesicular release of dopamine, thereby relieving TH from dopamine feedback inhibition. In contrast to the vector without *VMAT2*, injection of the four-gene vector into the rat striatum restored extracellular levels of dopamine and its metabolite dihydroxyphenylacetic acid to control levels and was able to support significant K(+)-dependent release of dopamine. Rats injected with the four-gene vector also showed higher levels of correction of apomorphine-induced rotational behavior even after 6 months.

ProSavin is a tricistronic lentiviral vector coding TH1, AADC, and GTPCH and was the first-in-man use of a lentiviral-based gene therapy vector for a chronic neurodegenerative disorder of the CNS. The long-term safety and tolerability of ProSavin was assessed in an open-label phase 1/2 clinical trial with 15 participants divided into three dosage groups (1.9 × 10^7^, 4 × 10^7^, and 10^8^ transducing units) with 12 month follow-up for at least 2 years.^23^^,^^24^ The administration was done bilaterally into the putamen, at a rate of 1 or 3 μL/min delivering in total 120–600 μL. The results showed a well-tolerated safety profile and led to improvement in motor behaviors in all patients, although this improvement was comparable with the placebo range. With the delivery of ProSavin, the transduced cells convert into dopamine-producing “factories.” As the vector does not encode dopamine trafficking genes, the mechanism of action of dopamine release has not been fully elucidated. The authors hypothesize that the dopamine produced diffuses into the extracellular environment, stimulating postsynaptic dopamine receptors in an autocrine or paracrine fashion. By optimizing the order of genes in the expression cassette and designing fusion variants of the genes, higher levels of dopamine production were achieved with the construct OXB-102 compared with that of ProSavin in transduced human primary neurons.^25^ Behavioral studies in a 1-methyl-4-phenyl-1,2,3,6-tetrahydropyridine (MPTP) macaque model of PD showed the effect of OXB-102 was similar to ProSavin, although with 5-fold lower concentration.^26^ The clinical trial for OXB-102 in patients with idiopathic PD was, however, terminated in 2022 and the results are yet to be published.

Partial reconstruction of the dopamine production pathway by AADC has also been investigated. The effectiveness of levodopa reduces as more dopaminergic neurons are lost over time. It has been shown that AADC gene therapy alone enhances levodopa response in PD patients after AAV2-AADC injection into the striatum by converting levodopa into dopamine.^27^^,^^28^ In a phase 1 trial, the safety, putaminal coverage, and AADC enzyme activity was assessed in 15 subjects with moderately advanced PD.^27^ The trial had three cohorts of five patients treated by simultaneous bilateral administration of AAV2-AADC (VY-AADC01) into the putamen in different doses: 7.5 × 10^11^ vector genomes (vg) in 450 μL, 1.5 × 10^12^ in 900 μL, and 4.7 × 10^12^ in 900 μL. Vector coverage was assessed by MRI with coadministration of a gadolinium contrast agent, and AADC enzyme activity was measured by ^18^F-DOPA PET after 5–6 months after treatment. It was shown that VY-AADC01 is well tolerated and AADC enzyme activity correlates with treatment dosage increase, improving Unified Parkinson’s Disease Rating Scale (UPDRS) III scores, motor fluctuations, and overall quality of life. Upstaza, an AADC gene therapy delivered by the AAV2-AADC system, was the first disease-modifying treatment for AADC deficiency approved in 2022 by the European Medicines Agency, and in 2024 by the US Food and Drug Administration under a different trade name Kebilidi. Although the loss of dopamine results from complex pathological processes, likely influenced by a combination of genetic and environmental factors, re-establishing components or the system as a whole would help to alleviate or slow the progression of symptoms.

Dopamine transporter deficiency syndrome (DTDS), also known as infantile parkinsonism-dystonia, is a rare disease without effective treatment caused by the loss-of-function homozygous mutations in the *SLC6A3* gene encoding the dopamine transporter (DAT). Lentiviral gene therapy with *SLC6A3* in an induced pluripotent stem cell (iPSC)-derived midbrain dopaminergic neuron DTDS model restored DAT activity and prevented neurodegeneration in DTDS patient iPSC-derived dopaminergic neurons. AAV2-SLC6A3 injection to the substantia nigra also ameliorated motor phenotype, lifespan, and neuronal survival in adult SLC6A3 knockout mice,^29^ demonstrating another example of gene therapy in the dopaminergic system.

### Controlling excitability with GAD

While PD is primarily characterized by impaired dopamine signaling, dysregulation of GABAergic neurotransmission has also been implicated in motor and non-motor symptoms.^30^ Studies of neuronal activity in MPTP-induced animal models of PD have reported abnormally increased activity in the subthalamic nucleus and globus pallidus.^31^^,^^32^^,^^33^^,^^34^ Subsequent research has shown that reducing the activity in the subthalamic nucleus, either by electrical stimulation, lesioning or treatment with GABAergic agonists can improve motor symptoms in humans and animal models of PD.^35^ Moreover, deep brain stimulation applied to either the subthalamic nucleus or globus pallidus, was found to improve motor function in PD patients that persisted for 3–4 years,^36^ suggesting that targeting hyperexcitability in PD leads to long-lasting clinical benefit.

Gene therapy utilizing a recombinant AAV vector-based system to deliver the glutamic acid decarboxylase (GAD) has been explored as a potential alternative therapeutic strategy to target hyperexcitability in PD (Figure 1B). This approach involves delivering the gene encoding GAD, which is responsible for converting glutamate to gamma-aminobutyric acid (GABA), to induce GABA production in the glutamatergic neurons of the subthalamic nucleus. Increased production of GABA in the subthalamic nucleus, and subsequently increased release of GABA at its terminal region in the substantia nigra, results in reduced neuronal firing in this region.^37^

A number of studies in animals have suggested that AAV-GAD is sufficient to rescue disease-relevant phenotypes without evidence of toxicity.^37^^,^^38^^,^^39^ A recombinant AAV-based (rAAV) therapy has been developed consisting of a 1:1 ratio of rAAV encoding two distinct forms of GAD cDNA: *GAD65* and *GAD67*. Transgene expression was driven by the CMV/CBA artificial promoter and the WPRE viral enhancer element. In early preclinical research conducted in rats, delivery of the virus into one side of the subthalamic nucleus was sufficient to induce *GAD* transgene expression. Compared with controls, where no significant increases were seen, delivery of rAAV-GAD65 in 6-OHDA parkinsonian rats led to a significant increase in GABA release in the substantia nigra following stimulation of the subthalamic nucleus. Delivery of rAAV-GAD65 was also associated with improved locomotor activity in parkinsonian rats, including reduced contralateral rotations, rescue of asymmetry in the head position bias test, and increased paw touching behavior, whereas delivery of rAAV-GAD67 led to a significant increase in total locomotor activity. Furthermore, rAAV-GAD65 delivery led to the increased survival of TH neurons in the substantia nigra and ventral tegmental area.^37^ In a later study, a rAAV2 vector using a JDK promoter led to high expression of *GAD65* and generated significant behavioral improvements in a rat 6-OHDA PD model. These changes were more pronounced than those observed when expression of *GAD65* was driven by the CMV promoter.^39^

Early-stage clinical trials showed promising results. The first-in-human study of rAAV-GAD began in 2002 (NCT00195143). The open label, safety, and tolerability trial recruited 12 patients with PD who were divided into three groups and treated with either a low-dose (1.0 × 10^11^ vg/mL), medium-dose (3.0 × 10^11^ vg/mL), or high-dose (1.0 × 10^12^ vg/mL) of rAAV-GAD infused via a cannula at a rate of 0.5 μL/min for 100 min into the subthalamic nucleus on one side of the brain. The rAAV-GAD therapy was based on previous work, where *GAD65* or *GAD67* expression was driven by the CMV/CBA promoter.^37^ The viruses encoding GAD65 or GAD67 were mixed in a 1:1 ratio before delivery. Significant improvements in motor UPDRS scores were observed at 3 months post surgery and remained evident until 12 months. These improvements in movement were mainly observed on the side of the body contralateral to the brain hemisphere that received the treatment.^40^ In a subsequent larger phase 2 placebo-controlled trial, 44 patients with advanced PD were recruited to receive bi-lateral rAAV-GAD injections into the subthalamic nucleus at a final vector concentration of 1.0 × 10^12^ vg/mL, infused via cannula at a rate of 0.23 μL/min for 2.5 h. Six months after rAAV-GAD delivery in patients who were off dopamine medication, motor UPDRS scores improved by 23.1%, which persisted 1 year post surgery. However, in the placebo-treated control patient group, an increase in motor scores of 12.7% was also observed.^41^ Therefore, while rAAV-GAD delivery improved motor scores by ∼10% when compared with the placebo-treated group, this was less of an improvement than typically reported with deep brain stimulation.^42^

In a subsequent double-blind placebo-controlled phase 1/2 study conducted in 2022, 14 PD patients were recruited and divided into three groups: a sham-treated group, a rAAV-GAD low-dose group (7.0 × 10^10^ vg), and an rAAV-GAD high-dose group (21.0 × 10^10^ vg). Primary outcome measures were safety and tolerability, but additional measures of motor function were taken at baseline, and at 12 and 26 weeks (NCT05894343). The study was completed in September 2024 and a press release reported that the treatment was safe, well tolerated, and a significant improvement in motor scores was observed in the high-dose group, but not in the sham or low-dose groups.^43^ Patients will continue to be followed up 5 years post treatment for monitoring and to evaluate the long-term durability of the treatment.

### Growth factor delivery

Trophic factors are naturally occurring proteins that support the growth, survival, and function of neurons, and therefore have the potential to protect or regenerate progressively damaged dopaminergic neurons in PD. Key trophic factors involved in neuroprotection include glial cell line-derived neurotrophic factor (GDNF), its close structural and functional analog neurturin (NRTN), cerebral dopaminergic neurotrophic factor (CDNF), mesencephalic astrocyte-derived neurotrophic factor (MANF), and platelet-derived growth factor-BB (PDGF-BB) (Figure 1C).^44^ These factors promote neuronal survival, enhance neuroplasticity, and may help to slow the degeneration seen in PD.

GDNF and NRTN both work on pathways through RET-induced activation of Nurr1, an intranuclear receptor that regulates the development of dopaminergic neurons by inducing expression of AADC, TH, DAT, and VMAT2.^45^^,^^46^ Intraputamenal infusion of recombinant GDNF was studied in a double-blind randomized phase 1 trial of 41 patients, where GDNF was administered every 4 weeks for 40 weeks.^47^ Interestingly, significant increase of ^18^F-FDOPA uptake was observed in the GDNF group, but no significant improvements were found in OFF state UPDRS motor score or any of the secondary or exploratory motor endpoints. In a recent study, increased GDNF expression was found in PD patient brain after 45 months of AAV2-GDNF infusion, along with TH-positive cell sprouting, catecholamine, and ^18^F-FDOPA signal increase, which were restricted to AAV2-GDNF-covered putaminal regions.^48^ However, GDNF did not reverse neurodegeneration of dopaminergic neurons and continued disease progression was expected in advanced PD patients. A phase 2 study started in mid-2024 with moderately affected PD patients receiving AAV2-GDNF gene therapy as an intraputamenal injection to measure its safety and efficacy, the results of which are yet to be published (REGENERATE-PD, NCT06285643).

Intrastriatal AAV2-neurturin gene therapy (CERE-120) showed neuroprotection of dopaminergic neurons in the rat 6-OHDA lesion model comparable with the effects of AAV2-GDNF.^49^ In an open-label human phase 1 study, 12 PD patients received CERE-120 bilaterally into the putamen in two dosage groups: 1.3 × 10^11^ and 5.4 × 10^11^ vg injected in four needle tracks on each side,^50^ and it was found to be safe and well tolerated. Although the treatment failed to change UPDRS motor scores after 12 months, secondary measures suggested that CERE-120 might have some delayed positive effects on motor functions. A broader, double-blind sham-surgery-controlled phase 2 study was initiated with 51 patients enrolled (24 treated with AAV2-neurturin while 27 had sham-surgery), and followed up for 24 months.^51^ There was no significant benefit of AAV2-NRTN therapy compared with the sham group. A long-term study was published significantly extending the safety data up to 5 years after intervention.^52^ Follow-up postmortem data from two PD patients who received CERE-120 8 and 10 years prior showed intense TH and RET expression in melanized nigral neurons. There was, however, no difference in the degree of Lewy body pathology in comparison with untreated control patients with PD, and α-synuclein aggregates were also detected in neurons that stained for NRTN, RET, and TH.^53^ There is no currently active clinical trial for NRTN.

CDNF and MANF are also emerging neurotrophic factors with potential for treating PD. They form a novel family of evolutionarily conserved, endoplasmic reticulum (ER) located and secreted NTFs, which promote dopaminergic neuron survival, reduce neuroinflammation, and protect against oxidative stress.^54^ Intrastriatal injection of recombinant CDNF was shown to improve motor function and preserve dopaminergic neurons in animal models of PD.^55^ Similarly, MANF has been shown to protect neurons from stress-induced damage and reduce cell death in PD models.^56^ A phase 1, randomized, double-blind, placebo-controlled clinical trial was done to demonstrate the safety and tolerability of monthly supplementation of human recombinant CDNF in PD patients by intraputamenal administration.^57^ Although CDNF was safe and well tolerated, there were no significant changes between placebo and CDNF treatment groups. Viral CDNF delivery in the 6-OHDA mouse model showed neuroprotective and functional restorative effects after injecting AAV8-CDNF^58^ or AAV2-CDNF^59^^,^^60^ into rat striatum by increasing TH levels, fiber density, and decreasing amphetamine-induced ipsilateral rotations. Interestingly, the intranigral transduction of both CDNF and MANF on a single lentiviral vector increased the number of TH-positive cells and the TH striatal fiber density as well, adding up their individual effects.^61^ In the MPTP mouse model, intrastriatal injection of AAV2-hCDNF was shown to prevent MPTP-induced motor impairment and reduce gait dysfunction as well as exerting neuroprotective effects by attenuating inflammation by effecting astroglia.^62^

PDGF is a cell membrane interacting protein, which induces cell survival and proliferative signal upon binding.^63^ PDGF was shown to improve the survival of embryonic TH-positive neurons *in vitro*,^64^^,^^65^ which led to a clinical trial in PD patients where human recombinant PDGF was administered via an implanted drug infusion pump.^66^ The study showed that PDGF-BB (dimer PDGF-B gene product) was safe and well tolerated in patients with moderate PD. Secondary outcomes, however, indicated similar improvement in all groups, including the placebo-treated patients. Viral delivery of PDGF could be a viable option to achieve long-term survivability to these neurons.

It is important to mention that, due to ethical considerations, only advanced-stage PD patients, who have already experienced significant loss of dopaminergic neurons, are eligible to participate in clinical trials. Notably, trials involving GDNF, NRTN, and CDNF have been conducted in patients 8–10 years after their PD diagnosis, when the selective loss of dopaminergic neurons is already at a late stage, making the neuroprotective effect harder to establish than it would be in patients treated within 5 years of diagnosis.^67^

### Gene therapy for recessive PD genes

PINK1 and Parkin are key proteins involved in mitochondrial quality control. PINK1 detects damaged mitochondria and accumulates on their outer membrane recruiting the E3 ubiquitin ligase Parkin, which is involved in the degradation of defective mitochondria through mitophagy (Figure 1D).^68^ Homozygous or compound heterozygous mutations on the *PRKN* and *PINK1* genes cause early-onset PD (EOPD), with slow progression, underlining the importance of mitophagy in the disease.

The gene *PARK2* encoding Parkin is one of the largest genes in the genome, with a 1.4 Mb locus located on the fragile chromosomal site FRA6E, prone to forming gaps or breakages.^69^^,^^70^ More than 170 different mutations have been identified making it the most frequent cause of EOPD. Mutated forms of Parkin show loss or reduced catalytic activity, aberrant ubiquitination profile, mislocalization, and/or degradation of the misfolded protein leading to the impairment of mitochondrial degradation.^71^^,^^72^ Mutations in *PARK2* are the most frequent known cause of EOPD (<40–50 years, 10%–20% worldwide, ∼50% of recessive familial forms).^73^ Patients with *PARK2* mutations have an early, slow disease progression without dementia in most cases,^74^ good responses to levodopa, although levodopa-induced dyskinesias developed in time and clinically indistinguishable from other patients with young-onset PD on an individual basis.^75^ Introduction of the correct *PARK2* gene was achieved in 6-OHDA-induced PD mice by AAV9-containing *PARK2* with a hydrophobic cell-penetrating peptide sequence (AAV-aMTD-Parkin), which led to the restoration of motor function.^76^ Four weeks after striatal injection of AAV-aMTD-Parkin into 6-OHDA PD mice, a significant motor function improvement of 102% was observed compared with the control group, as well as increase in TH expression in the treated mice. These experiments show the neuroprotective effect of Parkin, which could give rise to therapies aiming to treat PD patients carrying a *PARK2* mutation.

*PINK1* mutations are the second most common cause of recessively inherited EOPD, with more than 120 pathogenic mutations (missense, nonsense, frameshift) in *PINK1* identified.^77^ The majority of these are located on the serine/threonine kinase domain, indicating that disrupted kinase activity is a key factor contributing to the development of PD.^78^ It was shown in human neuroblastoma cells that PINK1 overexpression has a neuroprotective effect by strongly reducing both basal and neurotoxin-induced apoptosis.^79^ Overexpression of the wild-type PINK1, but not its PD-associated mutation G309D, was shown to alleviate α-synuclein-induced neurotoxicity in SH-SY5Y cells.^80^ Enhanced dopaminergic neurodegeneration was found in *Pink1* knockout mice,^81^ providing further evidence that mitophagy plays a very important role in PD. However, increasing PINK1 expression in other neurodegenerative diseases, namely Huntington disease and Alzheimer’s disease, has also proven beneficial,^82^^,^^83^ underlining the critical role of PINK1 in neuroprotection. PINK1 overexpression in a *Drosophila* model of HT, where mutant huntingtin was introduced, ameliorated mitochondrial spheroid formation and ATP levels, counteracting neurotoxicity.^83^ Intrahippocampal adenoviral delivery of PINK1 was studied in an Alzheimer’s model of transgenic mAPP mice that overexpress a human mutant form of APP, where transduction with AAV2-PINK1 reduced human amyloid-β levels by 65%–70% in the hippocampus significantly, increasing the clearance of damaged mitochondria by augmenting mitophagy.^84^

Loss-of-function mutations in DJ-1 represent the third most common genetic cause of recessive PD (Figure 1D).^85^ Several mutations on DJ-1 have been linked to PD, some with more penetrance than others.^86^ DJ-1 is a small 189 amino acid long redox-dependent molecular chaperone that inhibits α-synuclein aggregate formation and protects against reactive oxygen species and neurotoxins in a murine neuroblastoma line and in *in vivo* mice.^87^^,^^88^ AAV-DJ-1 was used in an MPTP PD mouse model, where its injection reduced MPTP-induced TH neuron loss significantly.^89^ DJ-1 expression was found to be sufficient to protect dopaminergic neurons against MPTP toxicity by modulating oxidative stress; however, DJ-1 did not increase dopamine levels and was found that it may also affect the synthesis, metabolism, and reuptake of dopamine.

Besides the three major genes attributed to EOPD with typical PD symptoms, there are other recessive genes including *PLA2G6*, *ATP13A2*, *HTRA2*, *FBX07*, *DNAJC6*, *SYNJ1*, and *VPS13C*, all of which might also be suitable for gene complementation therapy.

### Supporting lysosomes by *GBA1* delivery

Mounting evidence suggests that lysosomal dysfunction plays a key role in PD pathogenesis.^90^ Perhaps the most compelling evidence comes from a recent genetic study, which reported that >50% of individuals with PD carry one or more genetic variants linked to lysosomal storage disorders.^91^ Of these genes, *GBA1* has attracted particular interest, as mutations in the *GBA1* gene are one of the most common genetic risk factors for PD, increasing the risk of developing PD by 5- to 30-fold.^92^^,^^93^^,^^94^

A link between PD and *GBA1* mutations was first noted in the 1980s following observations that individuals with Gaucher’s disease (GD), a lysosomal storage disease caused by *GBA1* mutations, developed parkinsonism.^95^ The *GBA1* gene encodes the enzyme glucocerebrosidase (GCase), which has essential roles in lysosomal function through the hydrolysis of glucosylceraminde (GluCer) to ceramide and glucose in the lysosome (Figure 1E). Loss of Gcase leads to accumulation of GluCer, glucosylsphingosine, and other glycolipids leading to toxicity and inflammation. GCase has also been hypothesized to directly or indirectly impact α-synuclein aggregation. Given this, increasing GCase activity is considered a viable therapeutic strategy to improve lysosomal function and limit α-synuclein aggregation.^96^

GCase enzyme replacement therapy has been used as a treatment for GD since 1991.^97^ However, the recombinant forms of GCase (imiglucerase, taliglucerase alfa, and velaglucerase alfa) are unable to cross the BBB, making these treatments unsuitable for the neurological manifestations of GD or as a viable approach for PD.^98^ Moreover, there is a need to identify treatments that provide more stable production of the enzyme over time. Viral vector-mediated delivery of *GBA1* directly into the midbrain provides an alternative way to circumvent problems with BBB penetration and the transient lifespan of recombinant enzymes.

Several studies utilizing experimental mouse models of GD and PD have demonstrated that restoring GCase activity through viral-mediated delivery of *GBA1* is sufficient to rescue α-synuclein pathology, reduce neuroinflammation and gliosis, and prevent cognitive and motor deficits.^99^^,^^100^^,^^101^^,^^102^^,^^103^ AAV was used as the viral vector for *GBA1* cDNA delivery, although in various serotypes including AAV9,^99^^,^^100^ AAV2/5,^101^ and AAV1.^102^^,^^103^ These studies also differed in their choice of promoter to drive transgene expression, using the CBA promoter,^100^ the GUSB promoter,^99^^,^^102^^,^^103^ or the neuron-specific promoter synapsin.^101^ The majority of studies injected virus directly into the brains of mice, including fetal intracranial delivery into the lateral ventricle,^99^ unilaterally into the striatum, substantia nigra, and hippocampus,^101^ and bilaterally into the striatum or hippocampus.^102^^,^^103^ However, intravenous delivery of AAV9 variants expressing *GBA1* have also been shown to increase GCase expression in the brain and rescue disease-relevant phenotypes, both in adult mice via tail vein injection,^100^ and in neonatal mice via superficial temporal vein injection.^99^

With intravenous tail vein delivery of a modified AAV9 variant (AAV-PHP.B-GFP), containing a novel AAV9 capsid that demonstrates superior neuronal transduction following intravenous delivery, transduction led to widespread expression of GCase across the peripheral and central nervous system including in the forebrain, midbrain, and cerebellum. Moreover, delivery of *GBA1* cDNA in the AAV9-PHP.B vector (GBA1-P2A-GFP) was sufficient to prevent the formation of insoluble α-synuclein deposits across multiple brain regions, including striatum, visual, motor, somatosensory, and cingulate cortices in a A53T-SNCA transgenic mouse model of PD.^100^ These findings support previous work demonstrating the ability of AAV9 variants to cross the BBB and suggest that non-invasive intravenous delivery has the potential to treat disorders of the CNS.^104^^,^^105^^,^^106^ However, the consequences of more widespread gene therapy, as opposed to targeted expression in the brain, must be thoroughly explored if systemic delivery is to become a viable therapeutic option.

Based on encouraging preclinical findings, three clinical trials, sponsored by Prevail Therapeutics, were designed to investigate the safety and tolerability of AAV9-GBA1 (PR001/LY3884961) in GD and PD. The PROPEL study (ClinicalTrials.gov, NCT04127578) is a phase 1/2a study that aims to evaluate the safety and tolerability of PR001 in individuals with moderate to severe PD who carry at least one *GBA1* mutation. The study, which began in 2020, enrolled 20 patients who were split into low- and high-dose groups (the exact dose is not disclosed), with PR001 administered via intracisternal injection. In the first year, the safety, tolerability, immunogenicity, biomarkers, and clinical efficacy measures will be assessed. For a further 4 years, the safety of PR001 will continue to be monitored as well as reporting on selected biomarker and efficacy measures. The two further clinical trials, the PROCEED (ClinicalTrails.gov, NCT05487599) and the PROVIDE study (ClinicalTrials.gov, NCT04411654), are small-scale phase 1/2 trials to evaluate the safety and efficacy of single-dose PR001 in individuals diagnosed with type 1 and type 2 GD, respectively. For the PROVIDE study, PR001 was also administered via intracisternal injection, whereas the PROCEED study, which is focused on patients with peripheral manifestations of GD, involves intravenous infusion of PR001. These clinical trials are all currently still ongoing, with results expected to be released by 2028 for the PROVIDE trial, 2029 for the PROPEL trial, and 2030 for the PROCEED trial.

Viral-mediated delivery of GBA1 is a promising therapeutic strategy to address haploinsufficiency associated with a reduction in GCase activity and substrate accumulation. However, additional lines of evidence suggest that gain-of-function mutant forms of *GBA1* may also contribute to pathogenesis in PD.^107^ Most pathogenic mutations in *GBA1* are missense mutations leading to misfolding of the enzyme GCase.^108^ It was hypothesized that accumulation of misfolded GCase in the ER leads to ER stress, subsequent activation of the unfolded protein response (UPR), and ER-associated protein degradation. The prolonged activation of these processes can result in the activation of apoptotic pathways and neuronal loss. Indeed, several studies have demonstrated retention of GBA1 mutant proteins in the ER and subsequent activation of the UPR.^109^^,^^110^ Alternative gain-of-function mechanisms of mutant GCase have also been proposed, including impaired α-synuclein degradation via blockade of chaperone-mediated autophagy.^111^ However, numerous studies have demonstrated that knocking down *GBA1* or inhibition of GCase activity in cellular and animal models is sufficient to drive α-synuclein pathology.^101^^,^^112^^,^^113^^,^^114^ These findings suggest that a combination of both loss-of-function and gain-of-function mechanisms may underlie pathological processes associated with increased PD risk. Given this, complementary strategies that target both loss-of-function and gain-of-function mechanisms of *GBA1*-associated PD may be crucial for the development of an effective therapeutic.

### Reducing α-synuclein expression

A link between mutations in the *SNCA* gene and PD was first reported in 1997.^115^ The *SNCA* gene encodes the synaptic protein, α-synuclein, which is thought to play an important role in controlling neurotransmitter release from presynaptic terminals. In subsequent studies, duplication and triplication mutations in the *SNCA* gene were further associated with PD,^116^^,^^117^ where these mutations result in significantly increased levels of α-synuclein.^118^ Interestingly, a gene dosage effect was observed, whereby individuals harboring triplication mutations demonstrated an earlier onset, more severe disease phenotype than individuals with duplication mutations.^119^ These findings prompted the hypothesis that elevated levels of α-synuclein contribute to the pathogenesis of PD; therefore reducing overall α-synuclein levels may be a viable therapeutic strategy to reduce downstream pathology and neurodegeneration (Figure 1F).

Several studies have investigated the potential of RNA molecules to silence *SNCA* expression through RNA interference.^120^^,^^121^^,^^122^ AAV2 has been used to deliver short hairpin RNA (shRNA) to the substantia nigra in a rotenone rat model of PD. Treatment led to a 35% reduction in α-synuclein levels, which was sufficient to attenuate progressive motor deficit and prevent loss of dopaminergic neurons, presynaptic terminals, and dendritic processes.^122^ Using a similar approach, AAV2-mediated shRNA-*SNCA* delivery to the substantia nigra was found to attenuate forelimb deficits.^120^ Moreover, presymptomatic delivery of AAV1 vectors expressing an artificial microRNA to reduce α-synuclein levels (miR-SNCA) has been shown to prevent motor impairments in PD mouse models. Mice that express the human A53T-mutant α-synuclein were injected with AAV1 vectors encoding either miR-SNCA or a miR-GFP control into the hippocampus. One month later, mice were injected with 1 μg of human multiple system atrophy brain homogenate to induce synucleinopathy. Mice injected with AAV1 vectors encoding miR-SNCA demonstrated α-synuclein knockdown and reduced the extent and severity of α-synuclein pathology and motor deficits compared with mice injected with control virus.^123^

Alternative approaches to reduce α-synuclein expression have involved altering DNA methylation and using transcription factors to reduce gene expression. Regarding the former, an all-in-one lentiviral vector for targeted DNA methylation editing within the *SNCA* gene has been developed. This system used CRISPR-deactivated Cas9 fused with the catalytic domain of DNA-methyltransferase 3A to target hypomethylated CpG islands within intron 1 of the *SNCA* gene. In patient-derived iPSC-dopamine neurons with *SNCA* triplication mutations, treatment with the virus led to downregulation of *SNCA* mRNA and α-synuclein protein, which was sufficient to rescue disease-related phenotypes including mitochondrial ROS production and cellular viability.^124^ An alternative approach has been to deliver zinc finger repressors (ZFRs) via an AAV vector to repress *SNCA* expression. In preclinical data presented in 2024, treatment with ZFRs was found to reduce *SNCA* mRNA in human iPSC-derived neurons. The authors also examined the effects of their ZFRs in a PAC *SNCA* mouse model, which expresses the full-length human *SNCA* sequence together with its upstream regulatory region on a mouse α-synuclein-null background. AAV delivery of ZFRs administered via tail vein injection was found to significantly reduce *SNCA* expression in the midbrain and thalamus of mice 6 weeks post injection.^125^

Therapeutic approaches that aim to reduce α-synuclein levels must, however, be considered with caution, as the total loss of functional α-synuclein has also been proposed to drive neurodegeneration in some studies. Dopamine neuron loss has been reported following AAV2-shRNA-mediated *SNCA* knockdown, suggesting that persistent repression of *SNCA* expression can lead to toxicity.^120^ In an attempt to limit toxicity, the shRNA was embedded in a pre-miRNA mir30 transcript, which was delivered via an AAV2/8 vector system into rat substantia nigra. The authors reported positive effects on forelimb behavior and substantia nigra dopamine neurons, but observed inflammation and reduced TH expression.^126^ These results were in keeping with previously published findings, where transient reduction of *SNCA* levels using AAV-mediated delivery of either shRNA or siRNA was found to induce dopamine neuron loss and inflammation.^127^^,^^128^ To evaluate the long-term safety and efficacy of viral-mediated α-synuclein knockdown, rats were injected with either AAV2-shRNA-*SNCA* or an AAV2-shRNA-control directly into the midbrain and monitored for 12 months following injection. Treatment with AAV2-shRNA-*SNCA* led to a >70% reduction in α-synuclein levels compared with AAV2-shRNA-controls and no clinical abnormalities were reported in either group, suggesting that high levels of α-synuclein knockdown could be tolerated. There was some loss of TH expression in nigral neurons following transduction with either the AAV2-shRNA-*SNCA* or AAV2-shRNA-control virus; however, there was no evidence of disruption to dopaminergic neuron projection or neurodegeneration.^129^

Taken together these studies suggest that α-synuclein levels can be reduced robustly and for prolonged periods using AAV-mediated delivery of shRNA or siRNA targeting the *SNCA* gene. However, whether α-synuclein knockdown is a safe and viable therapeutic strategy relies on addressing discrepancies observed between studies regarding toxicity. Several hypotheses have been proposed based on the use of different strains of rats used, different AAV serotypes (AAV2-based versus AAV2/5-based), different promoters driving expression of shRNA, vector doses, and infusion rates.^129^ A greater understanding of the source of the toxicity observed in these studies is required before treatment approaches to knockdown α-synuclein can be pursued with confidence.

### Considerations and future research

Research into the development of gene therapies for PD has grown significantly over the last decade, driven by advances in our understanding of PD genetics, PD pathophysiology and the successful implementation of gene therapy in another neurodegenerative disorder, spinal muscular atrophy.^10^ However, current PD gene therapies have thus far demonstrated limited success. There are several biological, technical, and practical aspects surrounding vector design and the delivery of transgenes that must be addressed to optimize the therapeutic potential of gene therapy in PD.

Controlled, persistent expression of transgenes at physiologically relevant levels is required with precise regulation and control mechanisms in place to avoid mutations and insertional mutagenesis. To achieve this, one must consider the selection of the viral system, since different virus particles have diverse tropism to tissues or cell types, as well as dissimilar integration types, which define the length of transgene expression. The promoter driving the transgene is also important to take into account when engineering the vector, as some promoters are ubiquitously expressed, while others are cell-type specific (such as the neuron-specific synapsin 1 promoter),^130^ which could provide another layer of specific targeting. The promoter also impacts the expression over time: it has been shown that the CMV promoter has a tendency to be silenced by DNA methylation and histone deacetylation,^131^ while more physiological promoters are likely to last longer.

The capacity of the viral vector is a further consideration, as many viral vectors demonstrate limited transgene capacity only capable of delivering cDNA constructs. The HSV-1 amplicon system provides an opportunity to address this, with the potential for the delivery of transgenes of up to 150 kb, and multiple copies depending on the size of the transgene. This uniquely large capacity has been shown to support the delivery of large genomic DNA sequences expressing neurological disease genes encoding the complete gene locus comprising exons and introns together with the endogenous promoter in many cases.^132^^,^^133^^,^^134^ Delivery of the entire 135 kb *FXN* genomic DNA locus using a HSV-1-based amplicon vector into the mouse cerebellum led to high expression with little to no decrease in signal intensity even after 80 days.^135^ Moreover, HSV-amplicon-based delivery of a Parkinson’s gene to the nigrostriatal system has been shown to work effectively in mice,^136^ suggesting that such an approach could be used for delivering an entire locus to correct a familial mutation of PD genes.

A better understanding of treatment administration is required to find the most effective way for different gene therapies via standardized methods, as it still remains one of the greatest challenges. The majority of clinical studies have used cannulas to infuse virus directly into the putamen and/or midbrain; however, the rate of infusion and technology used differ across studies. Utilizing gadolinium-based contrast agents, such as those used in the AADC phase 1 clinical trial,^27^ would be immensely valuable to identify ideal delivery coverage for efficient viral infusion rates. Additionally, the optimal viral dose required to induce widespread anatomical coverage also remains to be determined, not just for different viral vectors but most likely different serotypes as well. While not yet examined clinically, intravenous delivery of gene therapies using AAV9 in mice has been shown to transduce several brain regions,^100^^,^^104^^,^^105^ suggesting that systemic delivery transfer through the BBB may be feasible. However, questions remain surrounding the long-term safety and efficiency of more widespread systemic delivery methods in humans. Furthermore, whether impairments to axonal transport, which is a key pathophysiological hallmark associated with neurodegenerative diseases including Parkinson’s disease,^137^^,^^138^ could disrupt vector delivery is currently unclear.

Future research may focus on strategies to insert a set of enzymes needed for a cascade or biological function, as in the case of ProSavin. Alternatively, the delivery of genes from multiple pathways with distinct functions could be beneficial, as shown in Wang et al.,^58^ where the combination of CDNF and AADC was used to treat severe lesions. HSV amplicon constructs hold great promise to deliver a series of genes encoding an entire pathway to treat certain cellular dysfunctions. While challenges remain, promising results from several early-stage clinical trials suggest that we can remain optimistic about the potential of gene therapy in the development of disease-modifying treatments for PD.

## Acknowledgments

The work is supported by a Sponsored Research Agreement from Replay.

## Author contributions

All authors planned the review. S.S. and E.C. contributed equally to the writing of the manuscript. R.W.-M. edited the manuscript.

## Declaration of interests

R.W.-M. is a consultant to and holds stock options in Replay.
